# Retroperitoneal Paraganglioma Presenting as a Chest Pain: A Case Report

**DOI:** 10.1155/2013/329472

**Published:** 2013-01-31

**Authors:** Parag Brahmbhatt, Pranav Patel, Atif Saleem, Rathi Narayan, Mark Young

**Affiliations:** ^1^Department of Internal Medicine, East Tennessee State University, P.O. Box 70622, Johnson City, TN 37614, USA; ^2^135 West Ravine Road, Suites 3 and 4 Kingsport, TN 37660, USA

## Abstract

Paragangliomas are very rare tumors derived from neuroendocrine cells of autonomic nervous system. Extra-adrenal paragangliomas account for only 10 to 15% of all paragangliomas and may present incidentally as a mass. Typical triad of fluctuating hypertension, headache, and sweating is not always present which makes the diagnosis difficult sometimes. Definitive diagnosis is usually made with histologic findings and surgery is the treatment of choice. We report a case of a 53-year-old male who presented with chest pain and vomiting.

## 1. Introduction

Paragangliomas are rare tumors arising from the neural crest tissue that develops into sympathetic and parasympathetic paraganglia throughout the body. Paraganglioma of adrenal medulla is known as pheochromocytoma while paragangliomas located outside of adrenal gland are broadly classified as extra-adrenal paragangliomas. Paragangliomas are also further divided into functioning and nonfunctioning based on their ability to secrete hormones. Functional paragangliomas have shown to secrete norepinephrine and nor-metanephrine resulting in clinical manifestations such as fluctuating or episodic hypertension, headache, and sweating. On the contrary, nonfunctioning paragangliomas are mostly asymptomatic and found incidentally as a mass. When nonfunctioning paraganglioma gets enlarged it presents as abdominal pain secondary to compression of surrounding organs. 

## 2. Case Report

A 53-year-old Caucasian male with past medical history of hypertension and hiatal hernia was brought to the hospital for evaluation of nausea and severe sharp chest pain. Chest pain was located retrosternally, radiating to both arm and back followed by episode of syncope.

Patient's vitals revealed tachycardia with heart rate of 108 per minute and tachypnea with respiratory rate of 24 per minute. Patient blood pressure was 119/70 mmHg and was maintaining good saturation on room air. Physical examination was within normal limits. Patients' blood work was unremarkable including negative cardiac biomarkers. Computerized Tomography (CT) scan of chest with intravenous (IV) contrast was negative for pulmonary embolism, thoracic aortic dissection, or aneurysm. Cardiac stress test was also within normal limit. 

On hospitalization day two, patient started complaining of sharp abdominal pain located over the right upper quadrant (RUQ). Ultrasound of RUQ was performed which showed 4 cm mass located adjacent to gallbladder without any gallstone or common bile duct (CBD) dilation. Endoscopic ultrasound (EUS) was performed to further evaluate the mass which revealed 3.5 × 3.4 cm solid, hypoechoic and heterogeneous mass near gall bladder without involvement of gallbladder, biliary tree, or liver and without any local lymphadenopathy. The common bile duct measured 9.5 mm and there were no stones or sludge in the biliary tree. Preliminary findings of fine-needle aspirates done during the EUS were consistent with low grade neoplasm, possibly vascular in origin with carcinoid tumor as a differential. 

**Figure 1:**
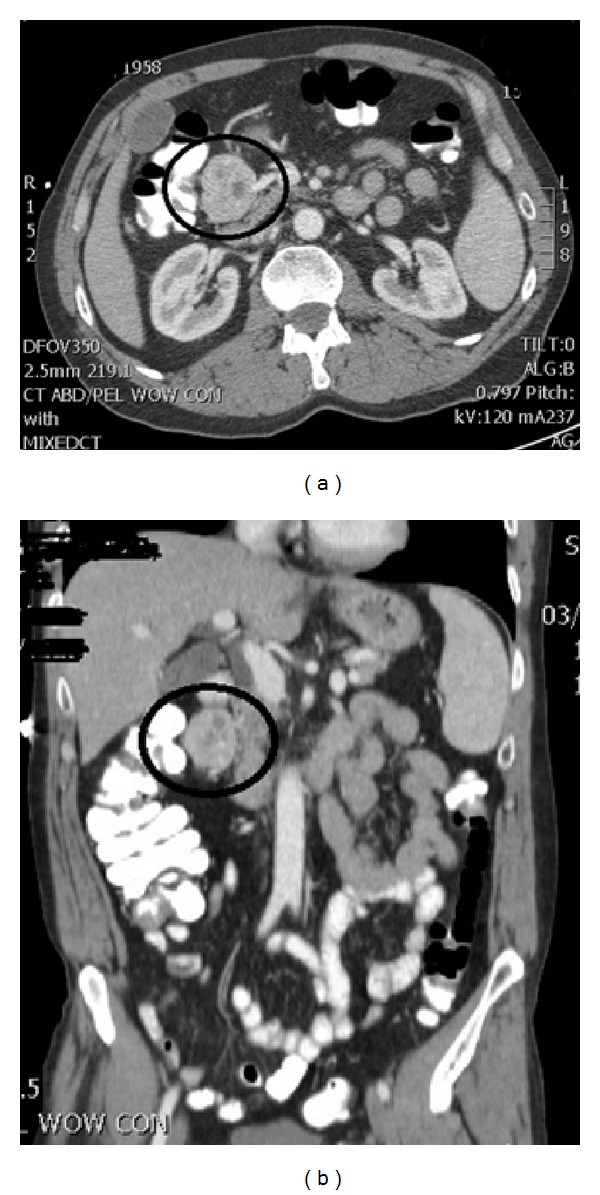
CT scan of abdomen and pelvis with contrast in (a) axial view and (b) coronal view showed well-defined 4.5 cm enhancing mass of mesocolon which was located anterior to descending duodenum, posterior to gastric antrum, and just medial to the hepatic flexure of colon.

CT scan of abdomen and pelvis with contrast was performed to further evaluate the mass which showed well-defined 4.5 cm enhancing mass of mesocolon which was located anterior to descending duodenum, posterior to gastric antrum, and just medial to the hepatic flexure of colon (Figure 1). It also showed several small cystic foci within the mass. 

Decision was made to proceed with laparoscopic surgical removal of mass which was later on converted to open laparotomy through right paramedian incision. Peroperatively, 5.7 cm × 3.8 cm mass was found within the C-loop just inferior to stomach, medial to duodenum, and on top of pancreas without involvement of any structure. The mass was excised entirely and was sent to pathology for further testing. 

**Figure 2:**
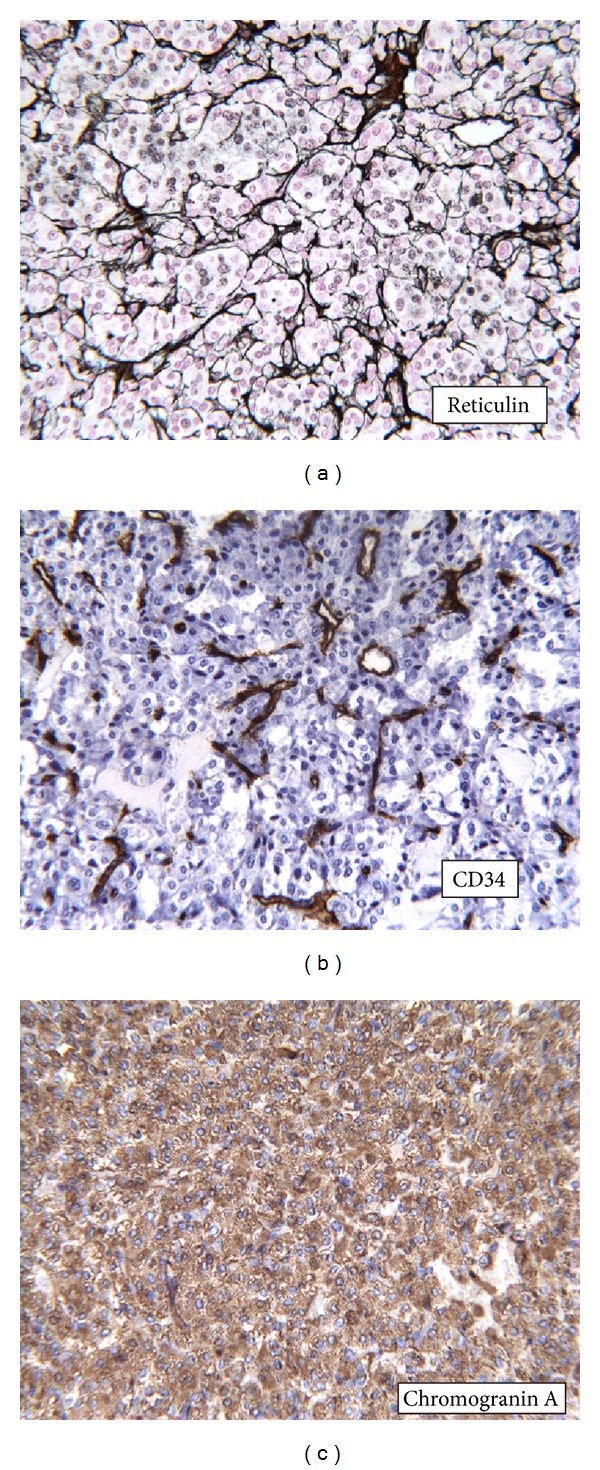
(a) Clusters of cells surrounded by reticulin fibrosis known as Zellballen arrangement, typical of paraganglioma. (b) CD34 immunostain marking the numerous small capillaries in the tumor. (c) Chromogranin A immunohistochemical stain.

Pathology revealed that tumor was comprised of a relatively uniform population of cells arranged in the clusters and these clusters were surrounded by reticulin fibrosis. This appearance is known as Zellballen arrangement, which is typical of paraganglioma (Figure 2(a)). The tumor was highly vascular in nature as highlighted by the CD34 immunostain (Figure 2(b)). Tumor cells were negative for epithelial AE1/AE3 cytokeratin and strongly immunoreactive for neuroendocrine markers CD56, chromogranin A (Figure 2(c)), and synaptophysin. Additional immunostains were performed to rule out carcinoid tumors and pheochromocytoma. 

Pathology also revealed that tumor was present within a fraction of a millimeter of the peripheral inked surgical resection margin. Positron emission tomography (PET) scan was performed for staging purpose which did not show any evidence of malignancy. Because of very close surgical margins of tumor cells, patient also received adjuvant radiotherapy. As there was no metastatic disease, chemotherapy was not provided. Patient is currently symptom free after 6-month followup and is closely followed by physicians. 

## 3. Discussion

Extra-adrenal paragangliomas account for 10 to 15% of all adult paragangliomas with an incidence rate of 2–8 cases per million people/year [1]. Age of onset is between 30 and 45 years with some literature suggesting male predominance, while other literatures suggest equal incidence between men and women [1]. 

Genetic mutation within the succinate dehydrogenase B unit (SDHB) and succinate dehydrogenase D unit (SDHD) are associated with increased risk for extra-adrenal paragangliomas. It has also been reported that incidence and prevalence of malignant paragangliomas are higher in patients with SDHB mutation [2]. There is also an association between extra-adrenal paraganglioma, gastrointestinal stromal tumor (GIST), and pulmonary chondroma which is known as the Carney's triad [3]. 

Abdominal paragangliomas are mostly retroperitoneal in location, accounting for 85% of all extra adrenal paragangliomas. The most common site for retroperitoneal paragangliomas is between the origin of inferior mesenteric artery and the aortic bifurcation known as organ of Zuckerkandl. Paragangliomas arising from jugulotympanic body are called chemodectomas, whereas paragangliomas originating from the carotid body are known as carotid body tumors. Paragangliomas located in the second part of duodenum are called gangliocytic paraganglioma [4]. 

As mentioned earlier different paragangliomas have different presentations based on location and ability to secrete hormones. Functional paragangliomas can be diagnosed based on presentation and subsequent laboratory investigation revealing elevated catecholamines and their metabolites in the blood and urine. Nonfunctional paragangliomas are mostly found incidentally or present as a mass with symptoms of surrounding organ compression. 

Recommended first imaging modality in evaluating extra-adrenal paragangliomas is magnetic resonance imaging (MRI) secondary to superior tissue characterization and absence of radiation hazards [3]. Computerized tomography (CT) scan also has sensitivity of around 90% for identifying extra-adrenal paragangliomas. On CT scan these tumors appear as soft-tissue masses with either homogenous enhancement or central areas of low attenuation. It appears as highly vascular structure with areas of intralesional hemorrhage and necrosis [5]. Metaiodobenzylguanidinescintigraphy (MIBG scintigraphy) has often been used as an imaging modality in the diagnosis of neuroendocrine tumors, but it lacks sensitivity for extra-adrenal paragangliomas. PET scan is another functional modality and has more sensitivity compared to MIBG scintigraphy [6].

Extra-adrenal paragangliomas also have potential to be malignant, although previously reported incidence of 10% is not accurate. It has been reported in the literature that around 20% of paragangliomas could be malignant with poor survival [7]. While histopathological findings are not much useful to differentiate between benign and malignant paragangliomas, extensive local invasion and distant metastasis to liver, bone, and lymph nodes have been used as indicators for malignancy [1, 5, 8]. 

Surgery with complete removal of mass either via laparoscopy or via traditional laparotomy is the treatment of choice for retroperitoneal paragangliomas owning to its malignant potential. Patient with metastatic disease will require adjuvant radiotherapy while chemotherapy is restricted to patients not accessible for surgery and resistant to radionuclide therapy [7]. 

Because of malignant potential and higher recurrence rate in paragangliomas, lifelong followup is usually recommended [6].
